# Site Distribution at the Edge of the Palaeolithic World: A Nutritional Niche Approach

**DOI:** 10.1371/journal.pone.0081476

**Published:** 2013-12-10

**Authors:** Antony G. Brown, Laura S. Basell, Sian Robinson, Graham C. Burdge

**Affiliations:** 1 Palaeoenvironmental Laboratories (PLUS), University of Southampton, Southampton, United Kingdom; 2 School of Geography, Archaeology and Palaeoecology, Queen’s University Belfast, Belfast, Northern Ireland; 3 MRC Lifecourse Epidemiology Unit, Southampton General Hospital, University of Southampton, Southampton, United Kingdom; 4 Institute of Developmental Sciences Building, Faculty of Medicine, University of Southampton, Southampton, United Kingdom; University of Oxford, United Kingdom

## Abstract

This paper presents data from the English Channel area of Britain and Northern France on the spatial distribution of Lower to early Middle Palaeolithic pre-MIS5 interglacial sites which are used to test the contention that the pattern of the richest sites is a real archaeological distribution and not of taphonomic origin. These sites show a marked concentration in the middle-lower reaches of river valleys with most being upstream of, but close to, estimated interglacial tidal limits. A plant and animal database derived from Middle-Late Pleistocene sites in the region is used to estimate the potentially edible foods and their distribution in the typically undulating landscape of the region. This is then converted into the potential availability of macronutrients (proteins, carbohydrates, fats) and selected micronutrients. The floodplain is shown to be the optimum location in the nutritional landscape (nutriscape). In addition to both absolute and seasonal macronutrient advantages the floodplains could have provided foods rich in key micronutrients, which are linked to better health, the maintenance of fertility and minimization of infant mortality. Such places may have been seen as ‘good (or healthy) places’ explaining the high number of artefacts accumulated by repeated visitation over long periods of time and possible occupation. The distribution of these sites reflects the richest aquatic and wetland successional habitats along valley floors. Such locations would have provided foods rich in a wide range of nutrients, importantly including those in short supply at these latitudes. When combined with other benefits, the high nutrient diversity made these locations the optimal niche in northwest European mixed temperate woodland environments. It is argued here that the use of these nutritionally advantageous locations as nodal or central points facilitated a healthy variant of the Palaeolithic diet which permitted habitation at the edge of these hominins’ range.

## Introduction

Two fundamental questions in Palaeolithic archaeology are: 1) do lithic distributions represent activity patterns? And 2) if so what can they tell us about Palaeolithic foraging strategies especially at the edge of the biogeographical range of hominins? Archaeologists accept that site distribution in later periods, both before and after the introduction of farming, can be used to infer subsistence strategies through spatial relationships with environmental factors both for mobile and sedentary societies [1]. Intuitively these relationships should be stronger for hominins prior to the innovation of food storage and durable receptacles. Such relationships should also be most marked at the edge of the geographical range of a species and the archaeological record suggests that hominins only occupied north-western Europe during cool to warm temperate conditions at least up until the last glacial cycle [2], [3]. For any research into Palaeolithic site distribution in North West Europe the starting point is that over 90% of Lower and early Middle Palaeolithic finds and sites in Britain and along the north European seaboard come from river gravel deposits [4]–[6], with the remainder mostly coming from raised beaches, caves or occasionally lakes and sinkholes (particularly dolines in this region). It is not surprising therefore that a strong taphonomic bias has been assumed given that lithic artefacts can be regarded as discoidal clasts and that many artefacts, particularly bifaces do exhibit signs of abrasion due to transport in fluvial environments [6], [7]. A second taphonomic factor is the visibility of these sites caused by the widespread practice of extracting sand and gravel (aggregate) by hand during the 19^th^ and early 20^th^ century. This fuelled an antiquarian appetite for lithics from gravel quarries including the payment of workers for ‘hand axes’ recovered, to the point to which some pits became more valuable for their artefacts than their aggregate [8]. It is very unlikely that rich sites were missed, given the large number of collectors, their particular preference for bifaces, and the large number of quarries operated principally for railway ballast and road construction. Several lines of research in geomorphology and archaeology over the last twenty years suggest that the artefact concentration particularly of bifaces at what has been termed ‘super-sites’ [9], is not primarily the result of taphonomic processes.

## Taphonomic Factors and Site Distribution

**Figure 1:**
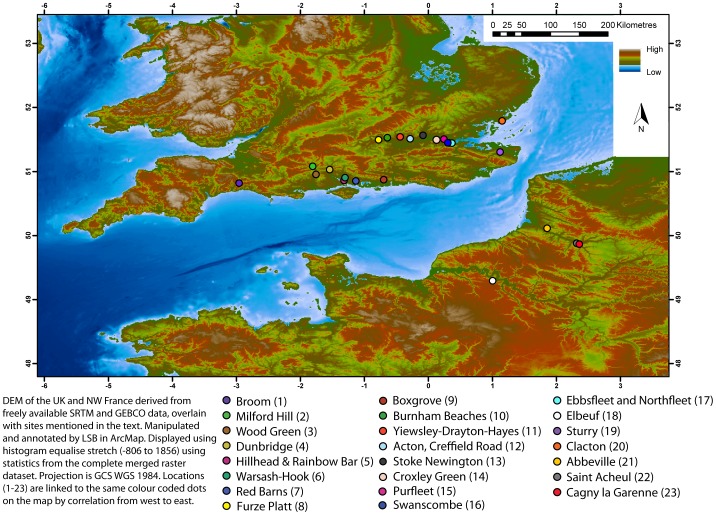
The distribution of Lower to Middle Palaeolithic (pre-MIS 5) sites in Britain and Northern France with over 500 bifaces (including roughouts) and other key sites referred to in the text.

**Figure 2:**
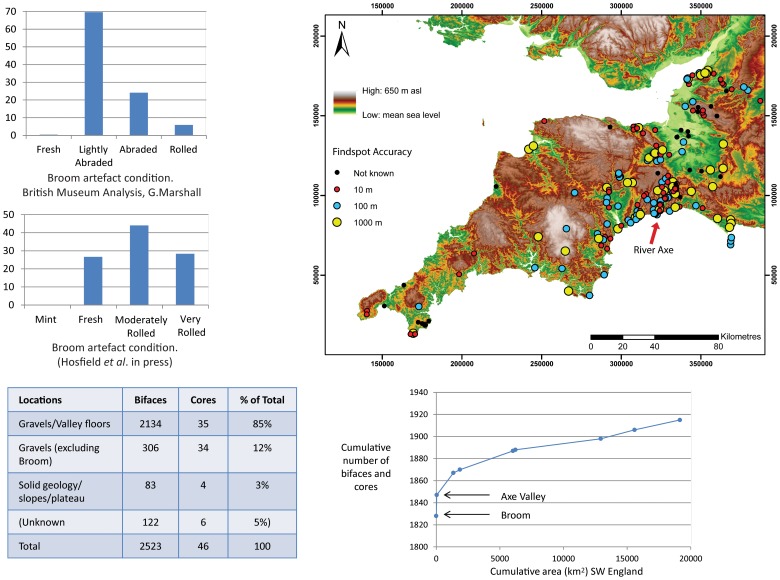
Palaeolithic site and find distribution in SW England from PRoSWEB with the cumulative number of artefacts plotted against area as a measure of the variance of site richness and statistics for find location.

For the English Channel River region which is geologically continuous, the first line of evidence comes from the characteristics of the site distribution and artefact density. In this study we have used bifaces (including rough-outs) rather than the entire number of lithics collected as bifaces represent one tool (rather than just knapping activity). Also because they were the most commonly collected artefact type, they should be conservative in statistical terms and the problem of between-site collector bias minimised. The frequency distribution of sites by biface number is highly skewed with most sites producing a small number of artefacts and only a very few sites producing large numbers (Fig. 1). In this study we have used data collected by Wessex Archaeology between 1993 and 1997 as part of The English Rivers Palaeolithic Survey and only that from single quarries (or gravel pits) or adjacent quarries. Although there are problems defining site size there are fewer than 25 Lower to early Middle Palaeolithic (pre-MIS 5) sites in the English Channel region that have yielded over 500 bifaces This pattern holds at a variety of spatial scales as in southwest England one site (Broom) has produced 96% of the bifaces from the region (20,000 km^2^). Research focussed on this region involving the location and enumeration of all known artefacts in museum collections has allowed calculations of the relative number recovered from valley floors and slopes (Fig. 2). The result is a valley/slope ratio between 26∶1 and 5∶1. Moving further east along the southern coast of Britain into the reach of the palaeo-Solent catchment that falls within the county of Hampshire, only 5 out of 281 sites have yielded 79% of all Lower and Middle Palaeolithic bifaces [10]. This is confirmed by the extreme rarity of finds on slopes and plateaus in this region, even in intensively field-walked areas. This spatial bias is illustrated by the cumulative plot of artefacts against area for the South West region (Fig. 2) which is analogous to a variogram with a high ‘nugget value’ and low range, and conforms to a typical hotspot-type distribution [11]. A very similar spatial pattern has been found for Palaeolithic sites in the Bose Basin, Guangxi, China [12] and in pre-agricultural archaeology in the USA [13]. Since the area covered by this study has been outside the maximum area of glaciation throughout the Pleistocene, glacial erosion cannot be the cause of this strong spatial bias, and although there has been intense and prolonged periglacial activity, solifluction deposits (locally known as ‘head’) very rarely contain any Palaeolithic artefacts although examples are known including the enigmatic site of Knowle Farm in Wiltshire [13], [14]. Another example is a biface from Boxgrove (unit 11) but this was found only 0.4 m away from an unmoved, refitting flake scatter [15], potentially indicating very limited transportation. The examination of many linear kilometres of exposures of head deposits in south western England as part of studies of Pleistocene environmental change has produced virtually no Palaeolithic artefacts [16]. In general solifluction deposits and tributary fluvial gravels deposited during cold marine isotope stages dilute concentrations in mixed and reworked alluvial formations as is clearly evident in the Axe Valley in South West England where background concentrations can be as low as 1 artefact per 200,000 m^−3^ of gravel [17]. Palaeolithic scatters do occur on interfluves in this region especially in association with clay-with-flints which is a Tertiary weathering deposit. However, even at sites with dense scatters of lithics, such as Wood Hill, Kent and Caddington and Round Green in Bedfordshire bifaces are rare [18], [19] and none approach the numbers seen at the floodplain super-sites.

A second line of evidence is artefact condition. Whilst many artefacts in river gravel deposits show signs of abrasion such as edge wear and rounding, many do not even those from the same site and stratigraphic level. At Broom using two different systems for recording lithic condition, between 27% and 70% are fresh to lightly abraded (Fig. 2.) [20]. Geomorphological research on clast rounding has further suggested that even those bifaces with ‘rolled to very rolled’ morphologies may not have travelled far, as the rate of rounding is proportional to clast shape [21] and their tips are frequently not broken. This contention is also supported by budget-based models of river terrace formation which suggest that the majority of gravels in terrace staircases in this region are derived from reworking of previous terrace deposits by lateral channel migration rather than downstream clast-abrasion [22], [23]. Even when the lithics are all abraded to heavily-abraded, as reported for Wood Green and Dunbridge (Fig. 1), the longitudinal occurrence of the high concentration is clearly peaked at 21-17 km upstream from the modern river mouth but downstream of the change in bedrock lithology [24]. The occurrence of both fresh and rolled bifaces in the same sedimentary bed, as at Broom and at Chard Junction (both in the Axe Valley) also suggest that the majority of the lithics have not travelled far and most probably are the result of local reworking of artefact spreads on the floodplain from adjacent gravel bars or ‘proximal contexts’ so although not truly in-situ they are still clustered near to the point of original deposition [25]. This also applies to probable lithic manufacturing sites such as Purfleet [26]. Such dense spreads or ‘pavements’ of lithics are known from several early Stone Age sites in east Africa such as Olorgesailie [27] and recent excavations at Rubirizi in Uganda [28]. The distinction between dense concentrations of artefacts in-situ or in proximal contexts, and the background lithic artefact content of fluvial gravel, is archaeologically critical as it allows such super-sites to be analysed as part of a behavioural rather than geomorphological distribution and permits meaningful ecological interpretation of site distribution.

## Site Distribution and Niche Reconstruction

The pre-MIS 12 hominin occupation of north-west Europe is only known from a small number of sites and may have been an Atlantic phenomenon restricted to mild to warm temperate climates in coastal areas [29]. Post-MIS 12 hominins occupied this region in all interglacials and some interstadials with the apparent exception of Britain in MIS 5e [30]. The distribution of the biface-richest post-MIS 12 but pre-MIS 5 sites in Britain and northern France shows a marked concentration in the lower reaches of river valleys (Fig. 1). The richest sites are defined as those with over 500 bifaces, which frequently occur together with other knapping products including cores and flakes (both prepared and non-prepared) of Acheulean and/or Levallois tradition. The sites can be grouped by catchments all draining into the English Channel or southern North Sea. In the Exe/Axe basin there is only one site at Broom [7], [19]; in the palaeo-Solent and East Sussex Plain system there are 7: Milford Hill, Wood Green, Dunbridge and Romsey, Hill Head/Rainbow Bar, Red Barns Warsash-Hook and Boxgrove [31]–[35]. Although large numbers of lithics have been found in the Bournemouth-Christchurch area they are both widely dispersed and come from a number of terrace units even at the richest site which is Barton on Sea [37]. Similar areas of high background densities occur elsewhere in the region such as Sturry in Kent [14]. In the Thames basin there are 11 sites, Furze Platt and Burnham Beaches in the Maidenhead area, Yiewsley-Drayton-Hayes, Acton and Stoke Newington in the West London area, Croxley Green in the Colne valley, Swanscombe [36], [4] and Clacton in the Thames estuary region [4], [14], [37] and the early Middle Palaeolithic sites at Purfleet, Northfleet, and Ebbsfleet also in the Thames estuary region [38], [2]. Across the English Channel the major sites are restricted to the Somme valley at the sites of Abbeville, St Acheul and Cagny la Garenne. Due to the low gradient and high tidal range of the Somme, Abbeville is only 3 km upstream of the present natural tidal limit (NTL) and St Acheul and Cagny are just over 40 kms upstream of the NTL. On the Seine, Elbeuf is actually at the present tidal limit [39]. In addition to these high-concentration sites there are a number of recently researched sites which have a small number of artefacts but good environmental and chronostratigraphic data such as Caours in the Somme Valley and La Celle in the Upper Seine-Yonne Valley [39]–[40]. Caours and La Celle have been included in the palaeoecological database along with Boxgrove as proxies for natural interglacial conditions and due to their comprehensive records.

The distance of the sites upstream of the NTL has been estimated from the corresponding interglacial relative sea level (IRSL) in order to approximate site position at the time of occupation. Estimation of the tidal limits in the last 4 interglacials (MIS 5e, 7, 9 and 11) have been made using the LRO4 stack [41] as a proxy for eustatic sea level adjusted for regional uplift [42] by regarding the associated raised beaches as indicating the interglacial sea level, and using the MIS 1 floodplain gradient (Table S1). This floodplain method was used in preference to the river terrace gradients as these are mostly cold-stage gravels and grade to low glacial sea levels [43], [22]. In the absence of any usable Pleistocene data, tidal frames have been assumed as similar to the present day. The results (Table S2) generally places the upstream sites slightly further above the NTL than they are today but the sites in the estuarine zone move closer to the NTL. The additional distances are relatively small being a maximum of 2 km in MIS 9 to a maximum of 23 km in MIS 7. Palaeoecological data suggests that some sites were closer to the NTL than predicted here, such as Swanscombe which has ostracod evidence that it was just above the tidal limit as well as a dolphin vertebra (Ingress Vale Pit) and a marine Gadidae bone [44], [45]. Although the estimates of site position in relation to IRSL NTL have only been made for the British sites it is likely that the Somme and Seine sites results would be similar to those from the north side of the English Channel. One uncertainty associated with these calculations is the possible coast to inland variation in uplift rates which could have resulted in gradients different from those used here. A second uncertainty is that occupation phases at some of the sites were probably not associated with peak interglacial sea levels such as Swanscombe and Cagny la Garenne. In these cases the estimation of their position within the valley can only be a minimum distance from tidal waters. Despite these considerations, it is clear from the calculations and the geomorphology of the fluvial systems entering the English Channel that during high or maximum RSLs in each of the last four interglacials all except two of these super-sites would have been within 40 kms of the tidal limit and most were considerably less. The only other exceptions to this pattern are Boxgrove (unit 4c) which was a coastal plain site close to a freshwater spring [46]–[47] and Hoxne which was close to an evolving lake shore/riverine setting [48], [49]. This 40 kms distance range for the majority of the sites places them within 1 to 2 days walk [50] of access to marine resources. This suggests that although marine resources may have supplemented diets they were not a major source of nutrient intake. It is also noticeable that 12 out of the 19 English sites come from tributary junctions reaches (Broom, Wood Green, Milford Hill, Romsey, Dunbridge, Warsash, Furze Platt, Burnham Beaches, Yiewsley, Croxley Green, Stoke Newington, Purfleet) in which there would have been a greater extent of floodplain and channel habitat. The concentration of these super-sites in this zone raises an important question. Were these lower reaches of valley floors particularly favourable locations or niches for hominin activities and visitation?

In order to test this hypothesis we have attempted to predict the likely distribution of nutrient resources during the last 4 temperate interglacials along a transect from valley floor to the low-altitude plateaus which characterise the topography of this region. This is based upon year-round occupation and the fundamental assumption that adequate nutrient intake is critical to hominin survival throughout the year and in all stages of the life-cycle including pregnancy and infancy. We also make the assumption that that the nutritional requirements of Middle Pleistocene hominins (*Homo heidelbergensis, Homo neanderthalensis*) were related to their phenotype and environment in a way which both maximised fitness and is comparable to their nearest living relative (NLR) (i.e. *Homo sapiens*). This is justified by the generally held view that hominin nutrient requirement and digestive physiology appears to be genetically conservative [50]–[52]. Although the floodplain offers a wide variety of resources (Table 1) adequate nutrient intake is critical for hominin survival and procreation. A third but less critical assumption, particularly pertinent to nutrient requirements, is that energy expenditure patterns in hominins were not unlike those seen today. Support for this assumption comes from studies of total daily energy expenditure in contemporary hunter-gatherers which suggests that metabolic rates were not significantly different when body mass is taken into account [50].

**Table 1:** Potential resources available on the floodplains of medium to large rivers in Britain and N France during interglacial and warmer interstadial periods of the late Pleistocene.

Floodplain Resource	Constitutive components
**Raw materials**	chert/flint/quartzite/andesite…as cobbles & hard rock exposures in gorges, also natural coppicing & saplings (due to successional growth on floodplains) suitable for spears & sticks
**Safety**	caves/rock shelters in gorge reaches, tree-throw pits (more common on floodplains due to restricted tree rooting depth)
**Shelter**	open grass/shrublands (maintained by grazing) in the channel zone & water barriers
**nutrients**	water: all structural & metabolic processes (flowing (non-stagnant) water) proteins: energy+ tissue development & repair (herbivore flesh, fish, USOs) carbohydrates: energy+ tissue development & repair (USOs, honey) lipids (fats): energy+ tissue development & repair (marrow, animal fats, fish) vitamins: metabolic function (see text) minerals: cell structure, metabolic processes (animal & plant foods, mineral springs & precipitates)

**Figure 3:**
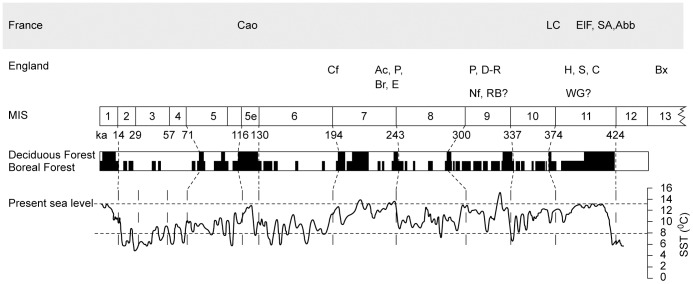
Vegetation reconstruction for southern England with SST from ODP 980 and approximate site chronology. Adapted from Stemerdink et(2010). Site code; Ac = Acton, Abb = Abbeville, Br = Broom, Bx = Boxgrove, C = Clacton, Cao = Caours, Cr = Crayford, D-R = Dunbridge-Romsey, E = Ebsfleet, ElF = Elbouf Fm., H = Hill Head, LC = La Celle, Nf = Newfleet, S = Swanscombe, SA = St Acheul, Wg = Woodgreen. Several sites have not been included as dating is too poor to assign them to an individual stage within the period (Furze Platt, Burnham Beaches, Yiewsley, Stoke Newington and Croxley Green).

**Figure 4:**
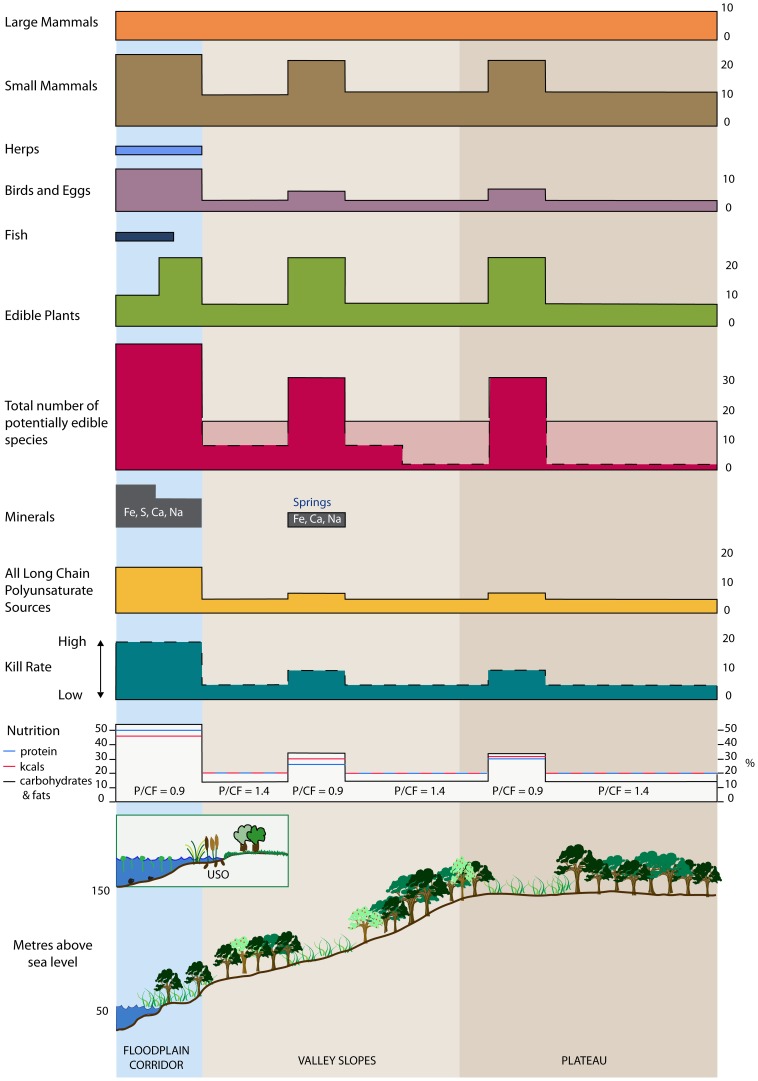
The nutriscape; a schematic representation of dietary diversity in a transect from valley floor to plateau top in the English Channel region. The scale (X axis) for each histogram is the number of species for each of the landscape zones. The variation in the total number of potentially edible species is to allow for more and less open forest cover.

Research by both archaeologists and Pleistocene scientists over the last fifty years has produced a significant record of both fauna and flora from both Palaeolithic archaeological sites and palaeoecological sites without any artefacts. These have included interglacial floral and faunal lists for the British Isles [53,45,54) and from the Netherlands [55] and France [56]. We have used these records (from 37 sites) to compile a list of potentially edible animals and plants that were present in the lowland landscape in southern Britain and northern France during temperate periods with a mean annual temperature of 7–12°C, namely MIS 11, 9, 7 and 5e (Fig. 3; Table S3) [57]. Further we have assumed that the only food processing technology available was roasting over an open fire. It is accepted that this cannot be a complete list and there may be some taphonomic bias to floodplain environments. An attempt has been made to counter this by including some species, which on ecological grounds probably were present but for which we have no fossil data as they are not floodplain species (e.g. Pignut; *Conopodium majus*). These are indicated in in the Supplementary Information along with the principal data sources and the food types with selected data in Table 2. The nutrient and energy values of these resources have been collated from a wide variety of sources but mostly nutritional databases (Canadian Ministry of Health, 1999; USDA National Nutritional Database; WHO Nutritional Landscape Information System). The edible status of these resources is taken from modern and historical observations and in effect excludes inedible grasses, woody tissues (except bark) and plants or animals known to be poisonous to humans [58]. Ecological classifications are taken from a wide variety of sources but particularly [59] for plants and [60] for mammals. As a first approximation to estimate nutrient intakes the landscape has been divided into 7 zones from the river to the plateau tops, which typically is a distance of 10 km and 150 to 200 m in relative relief (Fig. 3). The floodplain has further been divided into the river channels, including gravel islands and banks and the remainder of the floodplain including terraces. The rest of the landscape has been subdivided into forested slopes and plateau, and two open or large-gap environments. There are no continuous vegetation records that span the full period from this region but from fragmentary records and correlation with continuous sites to the south an approximate pattern of forest cover has been reconstructed by [61] which correlates reasonably well with human occupation (Fig. 3) [3]. Based on the typical mesocratic vegetation phase of interglacials in this region, forests would have been mixed temperate and deciduous and dominated by oak (*Quercus*), elm (*Ulmus*), lime (*Tilia*) and beech (*Fagus*) further to the south [62]. Although there was both systematic geographical and temporal variation during this period the temperate climate and areas of open grassland would have controlled the diversity of the shrub and herbaceous flora which includes the majority of edible species. It has been argued from a number of sites that the relative open-ness of interglacial floras was the result primarily of large herbivores and particularly the steppe mammoth (*Mammuthus trogontherii*) [63]. The distribution of large mammals (>30 kg) is assumed to have been even. However, this has been adjusted to reflect the relative ease of ambush predation and scavenging on the floodplain due to open conditions and the congregation of animals at drinking and bathing sites. The open nature of a significant part of the landscape is also suggested by the presence of horses (*Equus*) at nearly all sites. Late Pleistocene horses have a dental crown height ratio of over 5.5 and were therefore adapted to grazing rather than browsing [64]. There is also evidence from movement ecology that some of the species such as deer and horses are preferentially attracted to the forest edge, clearings and wetlands particularly during winter [65], [66]. Pending useable data on habitat use and relative kill rates it has been assumed that the availability/kill ratios (probability of encounter and kill rate) for clearings is 2∶1 and the floodplain 3∶1. Evidence-based estimates are extremely difficult but many of the species are only available to be killed (or gathered) in open habitats; and in terms of closed forest this would be in natural gaps and the floodplain. It is also likely that many large birds and herbivores would be at their most vulnerable when in, or at, water. Differential encounter or kill rates are not used in the summary nutritional values (Fig. 4) as they would primarily affect total intake per unit energy expended whilst the relative differences result from the species patterns. For small mammals ecological data has been used to assign species to principal locations and the herpetofauna and fish have been ascribed solely to the floodplain since these landscapes contain few persistent natural lakes. Although small in biomass, small mammals may have been important for prey-shifting (*sensu* [67]) in times when large mammals were scarce. Birds have also been classified according to habitat and the higher number of species in the floodplain corridor is a reflection of the high number of waterfowl recorded from Pleistocene sites in the region (e.g. Boxgrove [68]). Whilst there may be a bias here the greater ease of hunting waterfowl and egg collection has not been included although this would also be greater in the riparian zones. Edible plants are known from all ecological zones with similar assemblages from open areas and floodplains, although the nutritional value and ease of collection almost certainly varied. These species are then totalled to give a comparison of the zones revealing the greater diversity of edible animals and plants in the floodplain corridor and in clearings than in the intervening mixed temperate woodland. Certain potential nutrient sources have not been included particularly mollusca and insects. This is not because the authors discount them as potential sources, indeed both are potentially high in nutrients, but because of a lack of data on species-specific nutrient contents in this environment.

**Table 2:** Selected food groups from the database of interglacial flora and fauna for the southern England and northern France region with nutritional values.

Group/Type	Genera/species included	kcal per100 g	Proteinper100 g	Carb per100 g	Fat per100 g	Fibre per100 g	PUFA per100 g n3+n6?	Vit A IUper 100 g	Other important nutrients per 100 g
large herbivore (muscle)	**bison** rhino, bear,	223	18.7	0	15.9	0	0.75	0	Cholesterol (83 mg)
deer spp.	(red deer (venison)	111	22	0.2	2.6	0	0.35	0	Iron, Calcium
Marrow	caribou	786	6.7	0	84	0	0.25	240	Fat (86 g)
Marrow	beef/*Aurochs*	900	11	0	95	0	0.25	0.26	Fat (96 g), Sodium (139 mg)
Horse	domesticated horse	133	21	0	4.6	0	0.65	0	Cholesterol (52–63 mg)
small mammals	**rabbit**	114	22	0	2.3	0	0.45	0	Cholesterol (28 mg)
amphibians	**frogs**, toads.	73	16.4	0	0.3	0	0.10	50	
birds/goose/duck	**wild duck**, geese, water fowl	211	17.4	0	15.2	0	2.02	88	Cholesterol (68 mg)
eggs/duck eggs	**chicken** *, geese, ducks,	347	17.6	0	29.4	0	1.41	1900	Potassium
fish/eel	**eel**, stickleback, coarse fish	657	65.8	0	41.6	0		124	Potassium, Phosphorus
fish/salmon	**salmon/trout**	141	19.9	0	0	0	1.50	280	Calcium
snails	**raw snails**	90	16.1	2	1.4	0	0.25	100	Iron, Calcium
hazel nuts	**hazel nuts**	655	16.3	6	63.3	7.7	6.45	34	
aquatic plants (seeds)	**yellow waterlily**	361	7.9	80	0.1	19	0.16	0	Niacine (4.2 mg), VE (35.6 mg), Manganese (0.94 mg), Zinc (6.3 mg), Magnesium (86 mg)
aquatic plants (Tubers)	***Typha*** *, Phragmites*, *Trapa natans*(fruits), pignut	80	7.7	79.1	4.9	3.3	0.339	2	VC (42%), Folate, Calcium (50 mg)
berries/fruit	blackberries, **cranberry**	52.6	1.3	13.1	0	5.0	0	165	Potassium
leafy veg/	**spinach**, comfrey, scurvy grass.	24	2.9	3.6	0.4	2.2	0.16	9377	VC, VK, Folate, Manganese, Magnesium
aquatics leaves	**watercress**	11	2.3	1.3	0.1	0.5	0.03	3191	Calcium, Folate
salad/lettuce	edible leaf plants	13	1.3	2.6	0	1.3	0	469	VC (2.6 mg), Folate (27 mg)
honey	bees nests	309	0	85.7	0	0	0	0	Manganese (0.9 mg)
*EAR(Adult male )*	*modern diet*	*1900 kcal*	*56 g day*	*125 g day*	*30 g day*	*38 g day*	*1.6* *g day*	*3* *g day*	*Calcium (800 mg day), Iron (6 mg day), Vit C (75 mg day), Vit.B12(2 µg day), Niacine (12 mg day), Folate (120 µg day), Phosphorus (580 mg day), Magnesium (330 mg day)*

The animals and plants in bold have been used to estimate the nutrient values in the table.

indicates a taxa not present in the Early-Middle Pleistocene but used as a nutrient proxy. Carb, carbohydrate; EAR, estimated average requirements; VC, vitamin C; VK, vitamin K; PUFA, polyunsaturated fatty acids;

## Nutritional Diversity

There are over 50 nutrients required to sustain human life and diet has long been regarded as a driving force in biophysical, social and cultural evolution within the hominin lineage. Nutrients have classically been divided into 6 major groups (proteins, carbohydrates, lipids, vitamins, minerals, water). Whilst animal and plant tissues are the principal sources for all 6 groups the environment can directly supply minerals, including Fe, S, Ca, Na and Se with the principal source locations being springs and the floodplain in the form of Fe, S and Ca bio-precipitates. The relatively higher requirement for mineral intake during pregnancy has been associated with a desire for direct consumption of minerals [69], and mineral springs also attract mammals and plants which bio-accumulate these minerals, and metals in particular (metalophytes). Although most emphasis in reconstructions of Palaeolithic diets has been placed on protein intake, fat is the major energy reserve in hominids and can buffer food scarcity which would have been more common at the edge of the hominin geographical range. A fat-brain trade-off has also been proposed as an alternative to the gut-brain trade-off underlying the expensive-tissue hypothesis of brain enlargement [70], [71]. For over fifty years it has been recognised that there are certain nutrients which are essential for both human survival and reproduction. These are primarily micronutrients, vitamins and specific minerals, but also include specific amino acids and the essential polyunsaturated fatty acids (PUFA) which are obtained from plant sources, linoleic acid (LA) and α-linoleic acid (ALNA) [72]. LA and ALNA are essential in the diet because they are the respective precursors of longer chain PUFA including the *n*-6 fatty acid arachidonic acid, a precursor of prostanoids, and the *n*-3 fatty acid - docosahexaenoic acid (DHA) which plays a critical role in the development and function of the central nervous system [73] and which has been shown to modify epigenetic marks directly or indirectly [74]. Because of its critical role in the brain, DHA has been linked to human brain evolution [75]. Humans are poor converters of ALNA to DHA, although women do this more efficiently than men which has implications for the supply of DHA from mother to offspring [76], [77], and so preformed DHA derived from the foods at the land/water interface has been associated with the evolution of large brained hominins in Africa [78], [79]. Edible plants and animals rich in LA and ALNA, and animals rich in PUFA from plants (LA and ALNA), from meat (arachidonic acid) and fish (DHA) as groups have been totalled and show a marked increase in the floodplain corridor (Fig. 4).

For hominins including Neanderthals which have traditionally been seen as top carnivores [80] there is also a nutritional challenge in the protein ceiling in that protein intake must be balanced by carbohydrates and/or fats. If not so-called ‘rabbit starvation’ results due to the finite ability of the liver to regulate the rate-limiting enzymes that synthesise urea resulting in very high levels of ammonium ions and acidic amino acids in the blood [81]. This is known to have occurred in modern humans when they have been forced to rely entirely on fat-poor, wild animals [82]. We have used the plant and animal list to calculate the approximate balance of energy (kcals), protein and carbohydrates+fats (CF) as a percentage of the total values for the entire landscape diversity (all zones). As can be seen in Fig. 4 all three are highest in the floodplain zone, however, the floodplain corridor is the only part of the landscape where CF sources are potentially more abundant that protein sources. This is due to the availability of high CF sources including some high fat sources such as fish (particularly eels (*Anguilla)* which are available throughout the year), waterfowl and eggs; and high carbohydrate sources including plants particularly those with underground storage organs (USOs) such as reed mace (*Typha*), common reed (*Phragmites*), water chestnut (*Trapa natans*) and yellow water lily (*Nuphar lutea*). USOs have repeatedly been implicated in hominin evolution and particularly encephalisation and bipedalism in the Africa [83], [72], [84]–[85] although this has been challenged [86]. In order to maintain their protein:CF balance it is likely that hominins would have actively sought out high carbohydrate sources such as honey (86 g 100 g^−1^), aquatic seeds (80 g 100 g^−1^), berries (13 g 100 g^−1^), and hazelnuts (6 g 100 g^−1^). A similar propensity may have been displayed for high fat sources particularly bone marrow (84 g 100 g^−1^) and brain (70% fat and rich in arachidonic acid and DHA), but also hazelnuts (63 g 100 g^−1^), eel (42 g 100 g^−1^), birds eggs (29 g 100 g^−1^) and certain parts of selected mammals such as the tails of beavers (*Castor fiber*). Beavers are particularly interesting as they would have been ubiquitous damming all but the largest channels in this region, and at 20–30 kg they provide as much meat as a small deer whilst their tails can contain as much as 60% fat in the autumn and winter [87]. Presumably the same would apply on a larger scale with the large beaver-like rodent (*Trogontherium cuvieri)* which was present at Boxgrove, Swanscombe and Hoxne [88]–[90] but extinct after MIS 11 [91]. At Hoxne the remains of the otter (*Lutra*), beaver (*Castor fiber*), and waterfowl directly associated with the Acheulean lithics strongly suggested hominin exploitation, although no butchery marks were found on these species [88]. Bifaces are known to be extremely efficient butchery tools [92] and the emergence of particularly large and heavy bifaces may be related to their importance in marrow extraction in smashing the long-bones of large herbivores as well as for carcass processing. Likewise the high lipid content of brains including macaque and hominins [93] is also a potential driving force for the crushing of skulls and consumption of brain grey and white matter.

In these marginal temperate environments a similar problem arises with essential nutrients such as taurine, vitamins B6, B12, A and C, and folate. As can be seen from Table 2, viscera, especially liver, is particularly important in the supply of vitamin B6, B12, A and C as well as PUFAs, folate and some essential minerals. Fish, including eels are also a rich source of taurine, B6, B12 and vitamin A. Vitamin C is of particular importance as the body has limited capacity for retention (approximately 20 days) after which scurvy will arise if intake falls significantly below the daily requirement [94]. The principal source is herbivore liver, however, since vitamin C in liver is destroyed by cooking higher intakes are achieved if the liver is consumed raw [95]. Other sources are mostly seasonal such as berries and leafy vegetables. There are, however, year-round vitamin C sources within the riparian zone such as many species of the cabbage and carrot families including the wild cabbage (*Brassica oleracea*), watercress (*Nasturtium officinale*), wild carrot (*Daucus carota*), wild celery (*Apium graveolens*) and scurvy-grasses (*Cochlearia* spp.) all of which are found in disturbed floodplain environments and the upper estuarine zone. Also available year-round are sources of folate such as watercress (*Rorippa nasturtium-aquaticum*) and aquatic USOs such as water-chestnut (*Trapa natans*). USOs are particularly valuable in late fall and winter when they have their maximum energy values [58], [96]. The same is true for iodine for which there is an easily obtainable year-round source in non-acidic floodplains in the form of watercress although it would have varied with the geology and soils of the catchments [97]. Watercress is likely to have been particularly common around these sites as it benefits from nutrient enrichment in carbonate-rich waters and is tolerant of heavy grazing [59]. Analysis of the vitamin concentrations in these food sources highlights another potential problem in that there is very little vitamin E in the meat or indeed fish. Without adequate intake particularly during lactation there could be a substantial risk of oxidative damage to tissues (Table 3).

**Table 3:** Critical nutrients principal sources and some health implications.

Nutrient	Value/deficiency	Sources
PUFAs	Essential in brain development	Animal liver, brains, eyes, viscera, fish, some plants
Taurine	Important for brain function & eyes	Meat, fish
Vit B6	seborrhoeic dermatitis & if restricted canimpair fatty acid synthesis	fish, beef liver and other organ meats, some Underground Storage Organs
Vit B12	Damage to the brain and nervous system,cognitive disfunction, mania, psychosis, pernicious anemia.	liver, kidney, fish, shellfish
Fe, Zn	Anaemia, hypoplasty (eye sockets)/DNA synthesis…	Shellfish, liver, meat, nuts, seaweed
Iodine	thyroid function, goitre, cretinism, learning difficulties	Venison, watercress, leafy vegetables
Folate	Required to avoid birth defects (spinal)	Animal liver, leafy vegetables,
Vit A	Night blindness- erophthalmia, keratomalacia,	Animal liver, leafy vegetables, eggs, fish, berries
Vit C	to avoid scurvy (20 day residence time)	Liver, meat^1^, berries, leafy veg

^1^ only if eaten raw.

Vitamin D could also be a problem at these latitudes as it is only naturally present in a few foods such as fish and egg yolks. However, it must be noted this is difficult to assess as a constraint given the unknown hominin capacity for endogenously produced vitamin D from sunlight. The balance of Fe, I, Se, vitamins D and E and *n*-3 fatty acids are important in both metabolism and inflammation through their role as nuclear transcription factors and this balance is present in land-water ecosystems [85]. In relation to nutrient balance eels are a particularly interesting element of the ecology of the region in that they were ubiquitous, present all year-round, can move over land and would have been naturally attracted to kill and butchery sites on floodplains due to their ability to be able to detect blood in water. Furthermore eels are highly valuable nutritionally having almost equal quantities of protein and fat as well as being high in LCPs, vitamin A and key minerals (P and K). This and the use of other freshwater fish may be a cause of the greater dietary breadth estimated from isotopic values from early modern humans in Europe [99], [100] but it does not necessarily follow that this was a unique capability of modern humans. There is also a possible beneficial interaction here between the presence of beaver dams, which both facilitated river crossing (over the dams) and created ponds, but which also attracted both game and fish including eels.

**Figure 5:**
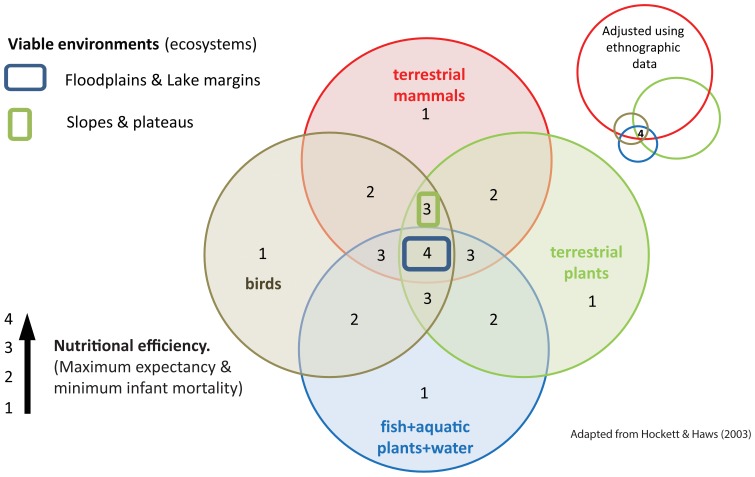
A representation of nutritional landscape ecology for a large temperate floodplain environment. Adapted from [124].

Taken together, the available energy, overall nutritional diversity, and availability of key micro-nutrients the floodplains provide the optimal location in this landscape through easy access to mammals of nearly all types, fish, waterfowl and edible plants including aquatic plants (Fig. 5). Unlike other zones the floodplain provides key potential resources for critical periods both during the year (e.g. for over-wintering [19]) and during critical periods in human life-cycles such as during pregnancy and breastfeeding. Other ecozones can also provide easy access to many of these resources, such as natural forest gaps and clearings, but none can provide such high on-site nutritional diversity. Nutritional diversity can be related to both high fecundity and lower rates of premature birth and infant mortality [100]. Sites in the wider parts of the floodplain such as downstream reaches and at confluence zones would have made beneficial bases or locations of concentrated activity from which other ecological zones could have been exploited and which would have formed hubs or central places in mobile hunter-forager circulatory patterns of seasonal mobility [100]–[101] over ranges of the order of 1000–2000 km^2^ [101]. This has been proposed as the pattern for Middle-Upper Palaeolithic site hierarchy and differentiation in the Rhone valley where the sites on the floodplain or valley floor are the long-term residential camps [102].

## Hunter-gatherer Diets, the Palaeodiet and Niche Construction

On the basis of animal bones, artefacts and stable isotope studies Palaeolithic hominins, and particularly Neanderthals, are generally regarded as top carnivores with meat making up the vast majority of their diet [9], [100]. As a result it has been argued that the palaeodiet was a low carbohydrate diet (c. 20–40%) but with essential nutrient requirements [103] and that the meat dependency would have increased with increasing latitude to a maximum in circum-Polar groups. However, studies of historical and contemporary hunter-gatherers only partially support this model. One of the problems has been that the vast majority of extant hunter-gatherer groups available for dietary studies are from either the low latitudes (tropics) or from high-latitudes (circum-Polar zone) with very few from the temperate zone and virtually none from the Old World temperate zone. Studies of Arctic hunter-gatherers have unsurprisingly shown levels of meat consumption reaching 90–100% by weight [104], [79]. However, it has been argued that there has been under-reporting of the non-meat component in these diets due both to seasonal variations and social factors [105]. Conversely studies of hunter gatherers in the tropics demonstrate that no exclusively vegetarian groups exist, and that the lowest meat intake is 20–30% [83]. Crucially a study of hunter-gathers in the Columbia Plateau [106] revealed a diet with approximately 30% animal food. Using any of these data the projected animal food intake for hunter-gatherers living at latitudes of this study (48°–53°) would be anywhere between 30% and 80% but probably not higher.

Most palaeoecological evidence of diet suffers from major problems of taphonomic bias particularly through the preferential preservation of bone including evidence of butchery. Isotopic analysis of bone collagen of Neanderthals suggests values of −21.8 to −19 and δ^15^N values of 8 to 12 [107], [97], [94]. Evidence from stable isotopic concentrations in hominin bones also has a dietary bias because there is a non-linear relationship between animal food intake and both δ^13^C and δ^15^N values [107], [108] and low-protein foods that may have been critical to survival are invisible in isotopic analyses of bone collagen [97]. The result is that on δ^13^C:δ^15^N plots differentiation between individuals who have 75% as opposed to 95% animal foods is very difficult. This is especially the case if their non-animal intake has been high in δ^15^N and δ^13^C and intake from other lower-protein foods cannot be measured. This problem of equifinality may be addressed over the next few years by studies of Sr:Ca and Ba:Ca ratios as both Sr and Ba decrease with increasing trophic level and are high in geophytes/USOs [83].

In summary the existing isotope literature may present a biased picture exaggerating meat consumption and under-estimating Palaeolithic consumption of plant food particularly critical, but low-volume, low-protein sources. This is supported by a few studies of independent sources of dietary information such as dental phytoliths. Data from Spy I and II in Belgium have revealed starch grains from USOs and grass seeds (Andropogoneae tribe) and from Shanidar III have produced starch grains of Type 1, probably from water lily [109]. Molar macro-wear studies [110] have demonstrated high dietary variability in Mediterranean evergreen habitats for both Neanderthals and early modern humans.

So far in this paper the concept of niche has been passive and rooted in twentieth century biology [111] under which environmental factors were seen to act on an organism in a particular place thereby creating its niche. However, modern concepts of the niche are not only multi-dimensional but also include the recognition that organisms, especially hominins, do more than passively survive in that hyperspace but modify their niches and those of other organisms through their metabolism, activities and choices [112]. The recognition of this reciprocal relationship lies at the heart of niche construction theory (NCT) as a key element of evolution [113]. One obvious feedback is the development of clothing allowing occupation further north and persistence in cool-temperate periods such as interstadials, also damping physiological selection in response to extreme temperatures [114]. More subtly the inhabitation and modification of the high-nutrient diversity niche described here may also have had a significant physiological and genetic legacy, analogous to, but very different from, later genetic legacies of agriculture. The nutrient diversity and opportunities to expand nutrient sources may well have propelled both behavioural change such as the increasing intake of marine foods and the development of fishing and of long-distance foraging seen in the later Middle and Upper Palaeolithic in this area [115], [116]. It may also have introduced an element of pre-adaptation to increasing diet breadth as epitomised by *Homo sapiens*. It is argued here that the result was that hominins located themselves in nutritionally optimum locations at the edge of their geographical range. Through repeated and/or prolonged occupation of those locales they altered them, probably creating adaptive responses in both the fauna and flora, and they were in turn altered by their constructed niche [113] particularly under migration restriction [117]. This is part of the ‘ebb and flow’ model of occupation of this region [3] and could also be a selective component of environmental remodelling that favoured adaptive plasticity or variability selection [116]–[118] resulting in occupation of the area during the last glacial cycle by both Neanderthals and modern humans well outside the climatic envelope considered in this paper.

## Conclusions

Based on our analysis of multi-disciplinary data we propose that the distribution of the richest Lower to Middle Palaeolithic archaeological sites in this part of northwest Europe is a real behavioural distribution reflecting hominin activity patterns rather than a taphonomic distribution. During the last four interglacials the location of the richest Palaeolithic sites in terms of biface densities is strongly biased to the lower reaches of river valleys and sites which were above NTL but in proximity to tidal rivers and estuaries. This may also have been the case earlier, as suggested by marine molluscs, barnacles and foramnifera at sites such as Pakefield and Happisburgh III [3] but analysis is prevented by the small number of sites and is complicated by the breaching of the Weald-Artois anticline creating the Straits of Dover and by major changes in fluvial geography [120], [28]. There are several reasons why lower reach of river valley floors would have been a favoured habitat in north west Europe including access to water, safety from predators, lowest river crossing points (natural rivers are shallow and frequently anastomose in these reaches facilitating crossing) and food resources. In this paper we have used a database of plant and animals resources known from Pleistocene sites in the region to compile a potential list of nutritional resources. Through ecological classification the nutrient landscape can be estimated and this shows a marked potential locational advantage for floodplain zones as opposed to the forested slopes, plateaus and even clearings. It is argued that this advantage may have included access to plants and animals which provided both essential energy and macronutrients but also critical micronutrients which maintained population health and maximised reproductive success and may have increased cultural complexity [121]. Such Palaeolithic diets with an aquatic component have been implicated in ‘healthy aging’, an emerging concept in evolutionary nutrition which has as its mantra ‘we are what we eat, but we should be what we ate’ [122], [87]. It is possible these locations were perceived as ‘healthy/good places’ to which hominins returned on a regular, and prolonged basis, and may have been ‘marked’ by their assemblage of artefacts [123]. If these were important and revisited locations they provide nodal points in the Palaeolithic geography of north west Europe and support the contention that river valleys provided the nutrient-rich route-ways of exploration and utilization of Palaeolithic landscapes. A possible symbiotic interaction through niche creation on floodplains is postulated between hominins, horses, freshwater fish (particularly eels) and beavers. It is also proposed that this optimal pattern of occupation in the nutritional landscape coupled with low population densities was fundamental to the Palaeolithic diet that was successful in facilitating expansion of hominins outside their evolutionary homelands and the persistent, if episodic, occupation of the less productive higher latitude regions of the northern hemisphere for over half a million years.

## Supporting Information

Table S1 **Parameters used for the prediction of relative site distance from the natural tidal limit (NTL) during the last 4 interglacials.**
(DOCX)

Table S2 **Distances from estimated NTL during middle-late Pleistocene Interglacials.** Calculations have not been undertaken where the sites are on eroded coastal cliffs and for Boxgrove and Hoxne.
(DOCX)

Table S3 **Summary occurrence and ecological data from edible plants and animals recorded in Middle-Late Pleistocene interglacials from southern England and northern France.**
(DOCX)
