# Supplementary material for: Site Distribution at the Edge of the Palaeolithic World: A Nutritional Niche Approach
Source: PLoS One. 2013 Dec 10;8(12):e81476. doi: 10.1371/journal.pone.0081476 (PMC3858259; doi:10.1371/journal.pone.0081476)
Supplement: Table S1 — Parameters used for the prediction of relative site distance from the natural tidal limit (NTL) during the last 4 interglacials. (DOCX) [file pone.0081476.s001.docx]

| **MIS (age in Ka)** | **D^18^O** | **Eustatic Component (m)** | **Uplift component (South Coast) (m)** | **Uplift component (Thames Valley) (m)** |
| --- | --- | --- | --- | --- |
| **1 (0-11.7)** | 3.85 | 0 | 0 | 0 |
| **5e (110-130)** | 3.90 | +5 | 7.2 | -5 |
| **7 (186-245)** | 3.65 | -25 | 13 | 11 |
| **9 (303-339)** | 3.80 | -5 | 19 | 15 |
| **11(380-423)** | 3.90 | +5 | 24 | 30 |

Table S1. Parameters used for the prediction of relative site distance from the natural tidal limit (NTL) during the Holocene and last 4 interglacials. The uplift components relate to the altitudes of the sites above present sea level based upon raised beaches and river terraces.
