# Supplementary material for: Site Distribution at the Edge of the Palaeolithic World: A Nutritional Niche Approach
Source: PLoS One. 2013 Dec 10;8(12):e81476. doi: 10.1371/journal.pone.0081476 (PMC3858259; doi:10.1371/journal.pone.0081476)
Supplement: Table S2 — Distances from estimated NTL during middle-late Pleistocene Interglacials. Calculations have not been undertaken where the sites are on eroded coastal cliffs and for Boxgrove and Hoxne. (DOCX) [file pone.0081476.s002.docx]

| **Site** | **Geomorphic position** | **MIS1 (NTL)** | **MIS7** | **MIS9** | **MIS11** |
| --- | --- | --- | --- | --- | --- |
| Broom | tributary junction | 13 | 20 | **15** | 11 |
| Milford Hill | tributary junction | 39 | 59 | **52** | **49** |
| Wood Green | tributary junction | 26 | 46 | **39** | **36** |
| Dunbridge | tributary junction | 22 | 47 | **27** | 17 |
| Romsey, | tributary junction | 5 | 30 | **10** | 0 |
| Red Barns | cliff line | 1.6 | - | **-** | **-** |
| Hill Head | floodplain | 0 | - | - | - |
| Warsash-Hook | tributary junction | 1.8 | - | **-** | **-** |
| Boxgrove | fossil cliff line, spring | 8 | - | - | - |
| Furze Platt | tributary junction | 28 | 63 | **35** | 21 |
| Burnham Beaches | tributary junction | 24 | 59 | **31** | 17 |
| Yiewsley-Drayton-Hayes | tributary junction | 9 | 44 | **16** | 2 |
| Croxley Green | tributary junction | 17 | 52 | **24** | 10 |
| Acton | floodplain | -7 | -28 | **0** | -14 |
| Stoke Newington | tributary junction | -22 | -13 | **-15** | -29 |
| Purfleet | tributary junction | -45 | -10 | **-38** | -52 |
| Clacton | channel/floodplain | - | - | - | - |
| Swanscombe (Barnfield Pit) | floodplain just above NTL | -50 | -15 | -43 | **-57** |
| Hoxne | lakeside | 55 | - | - | **-** |

Table S2. Distances from estimated NTL during middle-late Pleistocene interglacials. Calculations have not been undertaken where the sites are on, or near, eroded coastal cliffs and for Boxgrove and Hoxne. Bold highlights the MIS the site is could date to given present chronological resolution. A negative distance indicates the site is downstream of the tidal limit.

| **English name** | **Taxa** | **MIS13** | **MIS11** | **MIS 9/7/5e/2-1** | **Ecol. Zone** |
| --- | --- | --- | --- | --- | --- |
| **Mammals** | | | | | |
| bear/extinct cave bear | Ursus/Ursus deningeri | B | S,H,Bn |  | U,Af |
| elephant | Elephantid sp.(3) | B | S,H,C,Esr | Pu,Tr,Str Ca,Ma,SG | U,Aw |
| horse | Equus ferus (caballus) | B | S,H,Ca,RB | Pu,Ca,M,SG | U,Afw |
| extinct horse/ass | Equus hydruntinus |  | H | Ca | U,Afw |
| extinct rhinoceros | Stephanorhinus hundsheimensis, Dicerorhinus | B | S, H,C,Esr | Pu,Ma | U,Afw |
| red deer | Cervus elaphus | B | H,C,Ca,Esr | Com. | U |
| fallow deer | Dama dama, Dama clactoniana | B | S, H,C,Bn | Com. | U |
| roe deer | Capreolus capreolus | B | H,Esr | Com. | U |
| extinct giant deer | Megaloceros sp | B | H,Ca | Se | U |
| bison or aurochs | Bison sp, Bos sp. | B | H,Ca,Esr | Pu,Ma | U,Afw |
| caprine | Caprinae | B |  |  | U |
| wild boar | Bos primigenius |  | C | Ca | U |
| mountain hare | Lepus timidus | B |  |  | U |
| rabbit | Oryctolagus | B | S, Esr,Bn |  | U |
| beaver | Castor fiber | B | S,H,Esr | Pu | A |
| giant beaver | Trogonthorium cuvieri | B | S,H,C |  | A |
| otter | Lutra lutra |  | H |  | A |
| macaque | Macaca sylvanus |  | H | Pu | U,W |
| squirrel | Sciurus sp | B |  |  | U |
| Lemmings | Lemmus sp | B | H |  | U |
| voles & shrews | Microtus/Arvicola/ Clethrionomys/Pliomys/Neomys | B (9 sp) | H,C,Esr,Bn | Pu,Ma,SG | U,A |
| mice/Dormice | Muscardinus/Eliomys/Sicista/Apodemus | B (5 sp) | Esr,Bn | Pu | U |
| frogs/toads | Rana/Bufo | B (6 sp) | H,Esr,Bn | Pu,Ha | A |
| hedgehog | Erinaceus | B |  |  | U |
| European pond terrapin | Emys obicularis |  | Bn | Com. | A |
|  |  |  |  |  |  |
| **Birds** | | | | | |
| whooper swan | Cygnus cygnus | B |  |  | A |
| greylag goose | Anser anser | B |  |  | A |
| mallard | Anas platyrhynchos | B | H |  | A |
| wigeon | Anas penelope | B |  |  | A |
| gaganey | Anas querquedula | B |  |  | A |
| teal | Anas crecca | B |  |  | A |
| tufted duck | Aythya fuliula | B |  |  | A |
| dabbling duck | Anas sp |  | S |  | A |
| Indet. duck | Anatidae |  | S,Esr |  | A |
| goldeneye | Bucephala clangula | B | S |  | A |
| grey partridge | Perdix perdix | B |  |  | U |
| snipe or Plover | Scolopacidae/Charadridae |  |  |  | U,A |
| wood pidgeon | Columba palumbus |  | S |  | U |
| moorhen | Gallinula chloropus | B |  |  | A |
| tawny owl | Strix aluco | B |  |  | U |
| swift | Apus apus | B |  |  | U |
| robin | Erithacis rubecula | B |  |  | U |
| hedge sparrow | Prunella modularis | B |  |  | U |
| starling | Sturnus vulgaris | B |  |  | U |
|  |  |  |  |  |  |
| **Fish** | | | | | |
| eel | Anguilla anguilla | B | C, Esr,Bn | Pu, | A,At |
| smelt | Osmerus eperlanus |  | C |  | A,At |
| three-spined stickleback | Gasterosteus aculeatus | B | H,C,Esr,Bn | Pu,Ha,SG | A |
| salmon/trout | Salmonidae | B | S, Bn | Pu | A,At |
| pike | Esox lucius |  | H, Esr,Bn | Pu, | A |
| bleak | Alburnus alburnus |  | C |  | A |
| tench | Tinca tinca |  | H,Bn | Pu | A |
| roach | Rutilus rutilus |  | H,C,Bn | Pu | A |
| rudd | Scardinius erythrophthalmus |  | H,C,Bn | Pu | A |
| dace | Leuciscus leuciscus |  | C | Pu | A |
| carp family | Cyprinidae |  | C,Esr |  | A |
| ruffle or perch | Gymnocephalus cernua or Perca fluviatalis |  | Esr,Bn | Ha | A |
| burbot | Lota lota |  | Bn |  | A |
| minnow | Phoxinus |  | Esr |  | A |
| Baltic sturgeon | Acipensa sturio |  |  | Pu | At |
| cod | Gadidae |  | S |  | M |
|  | | | | | |
| **Invertebrates** | | | | | |
| snails | Mollusca | B (34 sp.) | Com. | Com. | U, A |
|  |  |  |  |  |  |
| **Higher Plants (eaten raw)** | | | | | |
| hazel | Corylus | Cr… | H,MT,S,C,Esr | Com. | U |
| beech | Fagus sylvaticus | WR | H,MT | Com. | W |
| walnut | Juglans |  | H |  | W |
| blackberries | Rubus sp | Cr | H, C | Wr,Ha,Ma | U,Wc |
| raspberry | Rubus idaeus | Cr | ? | ? | Wc |
| cloudberry | Rubus chamaemorus | FN | ? | Hn,Ha | UB |
| dog rose | Rosa canina |  | C | WW,Ss | Wc |
| sloe/cherry | Prunus spinosa | Cr | H,C | WW | U,Wc |
| hawthorne | Cretaegus monogyna | ? | C | Str | Wc |
| bird cherry | Prunus padus | ? | ? | Se | U,Wc |
| crab apple | Malus sylvestris | P |  |  | U,Wc |
| bilberry/cowberry/ | Vaccinium vitis-idaea, |  | G,Ba |  | Wc |
| cranberry | Vaccinium oxycoccos, V microcarpum |  | Ba |  | A |
| water chestnut | Trapa natans | P,M | Q | Wort,Ha,Tr,Ha | A |
| reedmace | Typha sp | Cr | H,S | Com. | A |
| common reed | Phragmites communis | Cr, M | K | WW,St | A |
| oat grass | Arrhenatherum tubersum | ? | ? | H | U |
| water lily(s) | Nuphar lutea, N. alba | Cr | Com. | Com. | A |
| water parsnip | Pastinaca sativa | Cr | H | ? | A |
| comfrey | Symphytum officinale | WR |  |  | U,A |
| pennywort | Hydrocotyle vulgaris | Cr,K, |  |  | U,A |
| primrose | Primula vulgaris |  | G |  | U |
| scurvy grass | Cochlearia officinalis |  |  | F | C |
| dandelion | Taraxacum officinale | ? | H | ? | U |
| good King Henry | Chenopodium bonus-henricus | ? | ? | Wr | U,W |
| lambs lettuce | Valerianella locusta | P | C | WW | Wc |
| Salad burnet | Sanguisorba minor |  |  | Hr | Wc |
| Sorrel | Rumex acetosa. R acetosella | Cr | Com. | Com. | U,W |
| lady’s smock | Cardamine pratensis |  | Wo |  | U,A |
| watercress | Rorippa nasturtium-aquaticum | ? | ? | HT,Ma | A |
| greater stichwort | Stellaria holostea | Cr | ? | ? | W |
| siverweed | Potentilla anserina | (Gu) | (Gu) | Se | U,A |
| snow thistle | Sonchus oleraceus |  | G | Br | Wc |
| winter cress | Barbarea vulgaris |  | C | Wr,Hr | A |
| redshank | Polygonum sp. | ? | Cr | ? | A |
| scarlet pimpernel | Anagallis arvensis | ? | N | Wr | Wc |
| common thistle | Cirsium vulgare | P | C | WW,Hr | Wc |
| water mint | Mentha aquatica | Cr.. | Com. | Com. | A |
| white water lily | Nymphae alba | Com. | Com. | Com.Hn,Str | A |
| yellow flag | Iris pseudocaris | ? | ? | Wr,Hn | A |
|  |  |  |  |  |  |
| **Probable** |  |  |  |  |  |
| wild garlic | Allium ursinum |  |  | Ea,Hn | W,A |
| brookweed | Samolus valerandi |  |  | Hn | A |
| pignut | Conopodium majus |  |  | Hn | U |
| wild strawberry | Fragaria vesca |  |  | Hn | Wc |
| prickly lettuce | Lactuca spp. |  |  | Hn | Wc |
| pennycress | Thlapsi arvense |  |  | Hn | Wc |
| wild celery | Apium graveolens |  |  | Hn | A |
| red clover | Trifolium pratense |  |  | Hn | Wc |
| chickweed | Stellaria media |  |  | Hn | Wc |
| shepherd’s purse | Capsella bursa-pastoris |  |  | Hn | Wc |
| Lesser celadine | Ranunculus ficaria |  |  | Hn | U,W,A |
| rosebay willowherb | Epilobium angustifolium |  |  | Hn | W,Wc |
| goats beard | Tragopogon pratensis |  |  | Hn | Wc |
|  |  |  |  |  |  |
| **Possible** |  |  |  |  |  |
| great willowherb | Epilobium hirsutum |  |  | Hn | A,Wc |
| bulbous buttercup | Ranunculus bulbosus |  |  | Hn | Wc |
| wild carrot | Daucus carota |  |  | Hn | Wc,A |
| wild cabbage | Brassica oleracae |  |  | Hn | U |

Table S3. Summary occurrence and ecological data from edible plants and animals recorded in Middle-Late Pleistocene interglacials from southern England and northern France. Sites**:** , B = Boxgrove, Ba = Baggotstown, Br = Brandon, Bn = Barnham, C = Clacton, Ca = Cagny , Cr = Cromer , Ea = Earith, Esr = Ebbsfleet Southfleet Rd, F = Farnham, FG = Fugla Ness, G = Gort, Gu = Godwin (1977) site unreferenced, H = Hoxne, Ha = Hackney, HT = Hawks Tor, Hr = Histon Rd, Hn = Holocene native, K = Kimington, MT = Marks Tey, M = Mundersley, Ma = Marsworth, N = Nechels, P = Pakefield, Pu = Purfleet, Q = Quinton, RB=Red Barns, S = Swanscombe, Se = Selsey, St = Stone, Str = Strensham, SG = Stoke Golding, Tr = Trafalgar Sq, Wo = Wolvercote , WR = West Runton, Wort = Wortwell, Wr = Wretton, WW = West Wittering. ? = not recorded at species level but likely to be present or recorded as present by [53] but with no site named, com. = common/many sites. Ecol. Zones: U = ubiquitous, open ground, disturbed ground, W = woodland, A = aquatic/wetland, UB = upland bogs. Subscripts, c = clearings, f = for feeding (incl. grazing), w = high water requirement, t = tidal/brackish, M = marine.
