# Supplementary material for: Site Distribution at the Edge of the Palaeolithic World: A Nutritional Niche Approach
Source: PLoS One. 2013 Dec 10;8(12):e81476. doi: 10.1371/journal.pone.0081476 (PMC3858259; doi:10.1371/journal.pone.0081476)
Supplement: Table S3 — Summary occurrence and ecological data from edible plants and animals recorded in Middle-Late Pleistocene interglacials from southern England and northern France. (DOCX) [file pone.0081476.s003.docx]

| **English name** | **Taxa** | **MIS13** | **MIS11** | **MIS 9/7/5e/2-1** | **Ecol. Zone** |
| --- | --- | --- | --- | --- | --- |
| **Mammals** | | | | | |
| bear/extinct cave bear | Ursus/Ursus deningeri | B | S,H,Bn |  | U,Af |
| elephant | Elephantid sp.(3) | B | S,H,C,Esr | Pu,Tr,Str Ca,Ma,SG | U,Aw |
| horse | Equus ferus (caballus) | B | S,H,Ca,RB | Pu,Ca,M,SG | U,Afw |
| extinct horse/ass | Equus hydruntinus |  | H | Ca | U,Afw |
| extinct rhinoceros | Stephanorhinus hundsheimensis, Dicerorhinus | B | S, H,C,Esr | Pu,Ma | U,Afw |
| red deer | Cervus elaphus | B | H,C,Ca,Esr | Com. | U |
| fallow deer | Dama dama, Dama clactoniana | B | S, H,C,Bn | Com. | U |
| roe deer | Capreolus capreolus | B | H,Esr | Com. | U |
| extinct giant deer | Megaloceros sp | B | H,Ca | Se | U |
| bison or aurochs | Bison sp, Bos sp. | B | H,Ca,Esr | Pu,Ma | U,Afw |
| caprine | Caprinae | B |  |  | U |
| wild boar | Bos primigenius |  | C | Ca | U |
| mountain hare | Lepus timidus | B |  |  | U |
| rabbit | Oryctolagus | B | S, Esr,Bn |  | U |
| beaver | Castor fiber | B | S,H,Esr | Pu | A |
| giant beaver | Trogonthorium cuvieri | B | S,H,C |  | A |
| otter | Lutra lutra |  | H |  | A |
| macaque | Macaca sylvanus |  | H | Pu | U,W |
| squirrel | Sciurus sp | B |  |  | U |
| Lemmings | Lemmus sp | B | H |  | U |
| voles & shrews | Microtus/Arvicola/ Clethrionomys/Pliomys/Neomys | B (9 sp) | H,C,Esr,Bn | Pu,Ma,SG | U,A |
| mice/Dormice | Muscardinus/Eliomys/Sicista/Apodemus | B (5 sp) | Esr,Bn | Pu | U |
| frogs/toads | Rana/Bufo | B (6 sp) | H,Esr,Bn | Pu,Ha | A |
| hedgehog | Erinaceus | B |  |  | U |
| European pond terrapin | Emys obicularis |  | Bn | Com. | A |
|  |  |  |  |  |  |
| **Birds** | | | | | |
| whooper swan | Cygnus cygnus | B |  |  | A |
| greylag goose | Anser anser | B |  |  | A |
| mallard | Anas platyrhynchos | B | H |  | A |
| wigeon | Anas penelope | B |  |  | A |
| gaganey | Anas querquedula | B |  |  | A |
| teal | Anas crecca | B |  |  | A |
| tufted duck | Aythya fuliula | B |  |  | A |
| dabbling duck | Anas sp |  | S |  | A |
| Indet. duck | Anatidae |  | S,Esr |  | A |
| goldeneye | Bucephala clangula | B | S |  | A |
| grey partridge | Perdix perdix | B |  |  | U |
| snipe or Plover | Scolopacidae/Charadridae |  |  |  | U,A |
| wood pidgeon | Columba palumbus |  | S |  | U |
| moorhen | Gallinula chloropus | B |  |  | A |
| tawny owl | Strix aluco | B |  |  | U |
| swift | Apus apus | B |  |  | U |
| robin | Erithacis rubecula | B |  |  | U |
| hedge sparrow | Prunella modularis | B |  |  | U |
| starling | Sturnus vulgaris | B |  |  | U |
|  |  |  |  |  |  |
| **Fish** | | | | | |
| eel | Anguilla anguilla | B | C, Esr,Bn | Pu, | A,At |
| smelt | Osmerus eperlanus |  | C |  | A,At |
| three-spined stickleback | Gasterosteus aculeatus | B | H,C,Esr,Bn | Pu,Ha,SG | A |
| salmon/trout | Salmonidae | B | S, Bn | Pu | A,At |
| pike | Esox lucius |  | H, Esr,Bn | Pu, | A |
| bleak | Alburnus alburnus |  | C |  | A |
| tench | Tinca tinca |  | H,Bn | Pu | A |
| roach | Rutilus rutilus |  | H,C,Bn | Pu | A |
| rudd | Scardinius erythrophthalmus |  | H,C,Bn | Pu | A |
| dace | Leuciscus leuciscus |  | C | Pu | A |
| carp family | Cyprinidae |  | C,Esr |  | A |
| ruffle or perch | Gymnocephalus cernua or Perca fluviatalis |  | Esr,Bn | Ha | A |
| burbot | Lota lota |  | Bn |  | A |
| minnow | Phoxinus |  | Esr |  | A |
| Baltic sturgeon | Acipensa sturio |  |  | Pu | At |
| cod | Gadidae |  | S |  | M |
|  | | | | | |
| **Invertebrates** | | | | | |
| snails | Mollusca | B (34 sp.) | Com. | Com. | U, A |
|  |  |  |  |  |  |
| **Higher Plants (eaten raw)** | | | | | |
| hazel | Corylus | Cr… | H,MT,S,C,Esr | Com. | U |
| beech | Fagus sylvaticus | WR | H,MT | Com. | W |
| walnut | Juglans |  | H |  | W |
| blackberries | Rubus sp | Cr | H, C | Wr,Ha,Ma | U,Wc |
| raspberry | Rubus idaeus | Cr | ? | ? | Wc |
| cloudberry | Rubus chamaemorus | FN | ? | Hn,Ha | UB |
| dog rose | Rosa canina |  | C | WW,Ss | Wc |
| sloe/cherry | Prunus spinosa | Cr | H,C | WW | U,Wc |
| hawthorne | Cretaegus monogyna | ? | C | Str | Wc |
| bird cherry | Prunus padus | ? | ? | Se | U,Wc |
| crab apple | Malus sylvestris | P |  |  | U,Wc |
| bilberry/cowberry/ | Vaccinium vitis-idaea, |  | G,Ba |  | Wc |
| cranberry | Vaccinium oxycoccos, V microcarpum |  | Ba |  | A |
| water chestnut | Trapa natans | P,M | Q | Wort,Ha,Tr,Ha | A |
| reedmace | Typha sp | Cr | H,S | Com. | A |
| common reed | Phragmites communis | Cr, M | K | WW,St | A |
| oat grass | Arrhenatherum tubersum | ? | ? | H | U |
| water lily(s) | Nuphar lutea, N. alba | Cr | Com. | Com. | A |
| water parsnip | Pastinaca sativa | Cr | H | ? | A |
| comfrey | Symphytum officinale | WR |  |  | U,A |
| pennywort | Hydrocotyle vulgaris | Cr,K, |  |  | U,A |
| primrose | Primula vulgaris |  | G |  | U |
| scurvy grass | Cochlearia officinalis |  |  | F | C |
| dandelion | Taraxacum officinale | ? | H | ? | U |
| good King Henry | Chenopodium bonus-henricus | ? | ? | Wr | U,W |
| lambs lettuce | Valerianella locusta | P | C | WW | Wc |
| Salad burnet | Sanguisorba minor |  |  | Hr | Wc |
| Sorrel | Rumex acetosa. R acetosella | Cr | Com. | Com. | U,W |
| lady’s smock | Cardamine pratensis |  | Wo |  | U,A |
| watercress | Rorippa nasturtium-aquaticum | ? | ? | HT,Ma | A |
| greater stichwort | Stellaria holostea | Cr | ? | ? | W |
| siverweed | Potentilla anserina | (Gu) | (Gu) | Se | U,A |
| snow thistle | Sonchus oleraceus |  | G | Br | Wc |
| winter cress | Barbarea vulgaris |  | C | Wr,Hr | A |
| redshank | Polygonum sp. | ? | Cr | ? | A |
| scarlet pimpernel | Anagallis arvensis | ? | N | Wr | Wc |
| common thistle | Cirsium vulgare | P | C | WW,Hr | Wc |
| water mint | Mentha aquatica | Cr.. | Com. | Com. | A |
| white water lily | Nymphae alba | Com. | Com. | Com.Hn,Str | A |
| yellow flag | Iris pseudocaris | ? | ? | Wr,Hn | A |
|  |  |  |  |  |  |
| **Probable** |  |  |  |  |  |
| wild garlic | Allium ursinum |  |  | Ea,Hn | W,A |
| brookweed | Samolus valerandi |  |  | Hn | A |
| pignut | Conopodium majus |  |  | Hn | U |
| wild strawberry | Fragaria vesca |  |  | Hn | Wc |
| prickly lettuce | Lactuca spp. |  |  | Hn | Wc |
| pennycress | Thlapsi arvense |  |  | Hn | Wc |
| wild celery | Apium graveolens |  |  | Hn | A |
| red clover | Trifolium pratense |  |  | Hn | Wc |
| chickweed | Stellaria media |  |  | Hn | Wc |
| shepherd’s purse | Capsella bursa-pastoris |  |  | Hn | Wc |
| Lesser celadine | Ranunculus ficaria |  |  | Hn | U,W,A |
| rosebay willowherb | Epilobium angustifolium |  |  | Hn | W,Wc |
| goats beard | Tragopogon pratensis |  |  | Hn | Wc |
|  |  |  |  |  |  |
| **Possible** |  |  |  |  |  |
| great willowherb | Epilobium hirsutum |  |  | Hn | A,Wc |
| bulbous buttercup | Ranunculus bulbosus |  |  | Hn | Wc |
| wild carrot | Daucus carota |  |  | Hn | Wc,A |
| wild cabbage | Brassica oleracae |  |  | Hn | U |

Table S3. Summary occurrence and ecological data from edible plants and animals recorded in Middle-Late Pleistocene interglacials from southern England and northern France. Sites**:** , B = Boxgrove, Ba = Baggotstown, Br = Brandon, Bn = Barnham, C = Clacton, Ca = Cagny , Cr = Cromer , Ea = Earith, Esr = Ebbsfleet Southfleet Rd, F = Farnham, FG = Fugla Ness, G = Gort, Gu = Godwin (1977) site unreferenced, H = Hoxne, Ha = Hackney, HT = Hawks Tor, Hr = Histon Rd, Hn = Holocene native, K = Kimington, MT = Marks Tey, M = Mundersley, Ma = Marsworth, N = Nechels, P = Pakefield, Pu = Purfleet, Q = Quinton, RB=Red Barns, S = Swanscombe, Se = Selsey, St = Stone, Str = Strensham, SG = Stoke Golding, Tr = Trafalgar Sq, Wo = Wolvercote , WR = West Runton, Wort = Wortwell, Wr = Wretton, WW = West Wittering. ? = not recorded at species level but likely to be present or recorded as present by [53] but with no site named, com. = common/many sites. Ecol. Zones: U = ubiquitous, open ground, disturbed ground, W = woodland, A = aquatic/wetland, UB = upland bogs. Subscripts, c = clearings, f = for feeding (incl. grazing), w = high water requirement, t = tidal/brackish, M = marine.
